# Melanoma as Subsequent Primary Malignancy in Hematologic Cancer Survivors—A Literature Review

**DOI:** 10.3390/jcm13154501

**Published:** 2024-08-01

**Authors:** Salomea-Ruth Halmágyi, Loredana Ungureanu, Ioana-Irina Trufin, Adina Patricia Apostu, Simona Corina Șenilă

**Affiliations:** 1 Clinical Hospital of Infectious Diseases, 400000 Cluj-Napoca, Romania; h_salomea@yahoo.com (S.-R.H.); ioana.irina.trufin@gmail.com (I.-I.T.); adinna.apostu@yahoo.com (A.P.A.); 2 Department of Dermatology, “Iuliu Hațieganu” University of Medicine and Pharmacy, 400006 Cluj-Napoca, Romania; 3 Department of Dermatology, Emergency County Hospital, 400006 Cluj-Napoca, Romania

**Keywords:** melanoma, hematologic neoplasm, chronic lymphocytic leukemia, non-Hodgkin lymphoma, second primary cancer, skin cancer screening

## Abstract

The occurrence of second primary malignancies is becoming increasingly important among cancer survivors. Melanoma, an aggressive neoplasm originating from the melanocytes, is responsible for most skin cancer-related deaths. This review aims to explore the risk of melanoma occurrence as a second primary cancer after the most common subtypes of hematologic neoplasia, a malignant disease originating from myeloid or lymphocytic cell lineages. Chronic lymphocytic leukemia (CLL) and non-Hodgkin lymphoma (NHL) are among the most associated subtypes with melanoma development. We also discuss the underlying hypotheses that may explain the associations between these malignancies and the impact of melanoma on survival. The review emphasizes the importance of increasing awareness of melanoma risk in hematologic cancer survivors, as it can lead to prompt recognition, improved skin surveillance, and better survival outcomes.

## 1. Introduction

According to the Global Cancer Statistics (GLOBOCAN) [1], an estimated 20 million new cancer cases occurred in 2022, with almost 10 million cancer deaths, and the annual number of cases is estimated to increase by 77% by 2050. Although incidence follows an ascendant trend, survival rates are also increasing; for example, in the United States of America, it is estimated that the number of cancer survivors will increase by 24.4% by 2032 [2]. In the period 2013–2019, the relative 5-year survival of all cancers combined was 68.7% [3]. Lengthened life expectancy, the implementation of screening programs for early detection, and improved therapeutics might all contribute to the rising trend in cancer survival [4]. 

However, with improved cancer survival, the importance of second primary malignancies becomes evident. A second primary neoplasm is defined by the occurrence of a second tumor, different from the first malignancy, in a patient with a personal history of treated or untreated cancer and that is not due to recurrence or metastasis [5]. A large population-based study in the United States of America showed that a second primary neoplasia occurred in 1 out of 12 survivors of frequent cancers. Moreover, the same study highlighted that 55% of the patients who developed a second cancer died because of it [6]. 

Melanoma, an aggressive neoplasm of the melanocytes, is less frequent than other types of skin cancers, albeit it is responsible for the majority of skin cancer-related deaths [7]. Melanoma incidence has risen in the last five decades, especially in light-skinned populations, and globally, it is estimated that approximately 325,000 new cases have occurred in the year 2020. Moreover, melanoma incidence is projected to further increase by more than 50% by 2040 [8]. 

Due to the important proportion of localized cases at diagnosis, melanoma is characterized by a relatively high 5-year overall survival rate of 93.5%. However, the prognosis is not as favorable in the late-stage disease, with survival rates of 73.9% in stage III and only 35.1% in stage IV disease [9]. Therefore, an early diagnosis of melanoma is pivotal. 

Risk factors for melanoma include a fair skin phenotype; a high number of acquired melanocytic nevi; large congenital nevi; a personal and family history of melanoma; dysplastic nevus syndrome; intermittent intense sun exposure, especially at a young age; exposure to artificial ultraviolet light (UV); immunosuppression; having a positive personal history for other types of skin cancer; and genetic predisposition [10]. 

Large population-based studies showed the occurrence of melanoma as a second primary cancer after other primary cancers [11,12,13]. Likewise, survivors of melanoma are at an increased risk of developing second primary cancers at various sites [14]. 

Hematologic cancers are malignant diseases originating from myeloid or lymphocytic cell lineages. The incidence of hematologic neoplasia has been increasing since 1990, but survival trends have also followed an ascendant trend due to improved diagnostics and treatment [15]. Hematologic neoplasia is known to be associated with secondary immune deficiency due to both disease-related and treatment-related factors, which may partly explain this risk [16]. Other factors can also influence the risk of second cancers, such as the long-term toxicity of treatments applied for the first neoplasm, exposure to mutual environmental risk factors, and a shared genetic predisposition [17]. 

Several studies indicated a higher-than-expected rate of melanoma development following different subtypes of hematologic malignancies, especially after chronic lymphocytic leukemia (CLL) and non-Hodgkin lymphoma (NHL), among other common subtypes [18,19,20,21,22]. 

With this review, we aim to provide an overview of the risk of melanoma occurrence as a second primary cancer after the most common subtypes of hematologic neoplasia. Furthermore, we discuss the underlying hypothesis that could explain the associations between these malignancies and review the impact of melanoma on survival. 

An increasing awareness of melanoma risk in hematologic cancer survivors is crucial, as it can lead to improved skin surveillance, prompt recognition, and better survival outcomes. 

## 2. Materials and Methods

We performed a literature search in the databases PubMed, Scopus, and Web of Science using various combinations of MeSH terms, including “Neoplasms, Second Primary” [Mesh] or “Neoplasms, Second Primary/epidemiology” [Mesh], “Melanoma” [Mesh], and “Leukemia, Lymphocytic, Chronic, B-Cell” [Mesh], “Lymphoma, Non-Hodgkin” [Mesh], “Hodgkin Disease” [Mesh], “Bone Marrow Transplantation” [Mesh], “Multiple Myeloma” [Mesh], “Lymphoma, T-Cell, Cutaneous” [Mesh] and keywords including “melanoma”, “second primary cancer”, “chronic lymphocytic leukemia”, “Hodgkin lymphoma”, “non-Hodgkin lymphoma”, “multiple myeloma”, “myeloproliferative disorders”, and “primary cutaneous lymphoma”. In addition, we screened the references of the included articles to find additional relevant sources. No time restrictions were applied to the literature search. The literature search took place between December 2023 and April 2024. We included all relevant studies published in English and available as full text until April 2024. We excluded studies explicitly investigating the occurrence of second primary cancers after childhood cancer and studies evaluating the risk of second primary cancers after hematologic neoplasia that did not assess melanoma risk.

## 3. Results and Discussion

### 3.1. Melanoma after Chronic Lymphocytic Leukemia/Small Cell Lymphoma

Chronic lymphocytic leukemia (CLL) is the most frequent subtype of leukemia in developed countries, with a globally increasing incidence from 1990 to 2019 [23]. Chronic lymphocytic leukemia affects mainly older adults, with the highest incidence in individuals above 70 years of age, especially in high-income countries [24]. It is characterized by a clonal expansion of small mature B lymphocytes in the blood, bone marrow, and lymphoid tissue, often with an indolent clinical course. Small lymphocytic lymphoma (SLL) represents a part of the same spectrum, wherein the proliferation of the monoclonal B cells with the same morphology and immunophenotype as in CLL infiltrates predominantly the lymph nodes, liver, spleen, and other tissues [25]. In the last 30 years, there has been a growing amount of data suggesting that CLL/SLL patients are at an increased risk of subsequent primary non-hematologic cancers, which was further supported by large population-based studies [26,27,28,29]. Among the second primary cancers encountered in CLL, melanoma plays an important part. A summary of the relevant population-based cohort studies highlighting the risk of melanoma in CLL/SLL survivors is presented in Table 1. 

As highlighted in Table 1, the standardized incidence ratio (SIR) is significantly elevated for melanoma as a second primary cancer after CLL/SLL in all the presented large population-based cohort studies except for the study conducted by McKenna et al. [30]. Although the SIR was not significantly increased in this Scottish cohort study, it is noteworthy that there was a statistically significant increase in the risk of a melanoma diagnosis in the first two years of follow-up [30]. Other studies also reported an increased risk of melanoma after the diagnosis of CLL. Tsimberidou et al. [32] analyzed melanoma risk among 2028 CLL patients in a single institution study and compared it to the risk of melanoma in the general population from the Surveillance, Epidemiology, and End Results (SEER) data. The authors reported an increased observed-to-expected ratio of 6.17. Sayin et al. [33] also demonstrated a higher risk of developing melanoma in CLL patients (O/E ratio 8.22) in a multicenter retrospective study including 553 patients from Turkey. Based on the Swedish Family Cancer Database, Zheng et al. [34] reported 127 melanomas occurring in 18,407 CLL patients, with a relative risk (RR) of 3.22 (2.71–3.83). Falchi et al. [35] analyzed the risk of subsequent primary cancers among long-term survivors of CLL and demonstrated a significantly elevated risk of cutaneous melanoma with a SIR of 3.78 (2.16–6.14). 

The risk of melanoma after CLL/SLL is similar in men and women, as shown in Table 2. Regarding age, patients who were diagnosed with CLL/SLL at a younger age had a higher frequency of melanoma, according to some of the studies [27,28]. However, one study showed an elevated risk of melanoma only in patients diagnosed with CLL after the age of 50 years [30]. In contrast to the general population, several studies showed a more frequent occurrence of melanoma on highly sun-exposed sites, such as the head and neck regions [6,19,20] and the limbs [28]. 

#### 3.1.1. Factors Contributing to Melanoma Risk in CLL/SLL Patients

**Figure 1:**
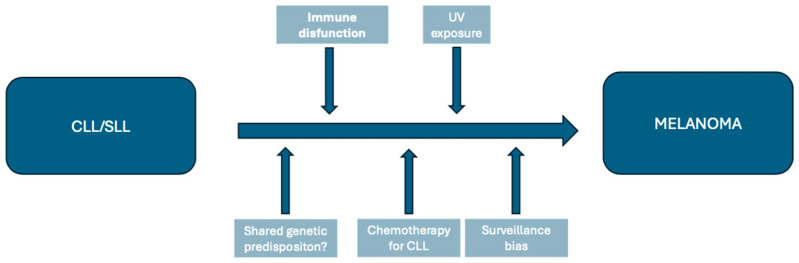
Factors that might contribute to the elevated risk of melanoma development after a diagnosis of chronic lymphocytic leukemia/small cell lymphoma.

Although it is not fully elucidated, multiple factors have been hypothesized to contribute to the excessive risk of melanoma in CLL/SLL patients, as illustrated in Figure 1. 

##### Immune Dysfunction

Several authors suggested that the increased risk of melanoma after CLL/SLL might be explained by the altered immune response, similar to the situation observed in organ transplant recipients [26,34,36]. Melanoma is an immunogenic tumor. The immune system has a critical influence on tumor development and prognosis. The role of the immune system in melanoma is supported by the possibility of spontaneous regression but also by the frequent presence of tumor-infiltrating lymphocytes in the tumoral tissue, mainly composed of CD8^+^T-cells, which are considered the most important effectors of the anti-tumoral response [37]. Immune dysfunction, as shown in the case of organ transplant recipients, not only carries a higher risk of developing melanoma but also a worse prognosis and higher mortality [38]. Similarly, patients with lymphoproliferative disorders present an immunocompromised state, affecting both the innate and adaptative immune systems, which may further be enhanced by the cytoreductive treatment [38]. 

##### Ultraviolet Exposure

Excessive sun exposure is a well-known risk factor for melanoma, but its relationship with CLL is controversially discussed. Although some authors suggest that UV light exposure might be a protective factor for CLL [39,40], a meta-analysis conducted by Lu et al. [41] failed to demonstrate a significant association between occupational sun exposure and the risk of CLL. Nevertheless, the harmful effects of ultraviolet light exposure on the skin may be accentuated in the context of immunosuppression. 

##### Genetic Predisposition

A shared genetic predisposition might also be partly responsible. 

The B cell leukemia/lymphoma 2 (BCL-2) protein family, with an essential role in apoptosis, has been known for a long time to play a crucial role in CLL development. The over-expression of BCL-2, an anti-apoptotic protein, has been demonstrated to be of central importance in CLL, which even led to the development of novel therapeutic agents [42]. Moreover, the BCL-2 family of proteins is over-expressed in melanoma, which has implications, especially in disease progression and therapeutic response. However, the role of these proteins in melanoma development is still under debate [43]. 

The Immunoglobulin Heavy-Chain Variable Region (IGHV) gene is an important prognostic marker in CLL, and the absence of mutations in this gene is associated with a poor prognosis [44]. An intriguing finding of the largest non-registry-based multicenter study was that melanoma development in CLL/SLL/MBL (high-count CLL-like monoclonal B-cell lymphocytosis) patients was associated with unmutated IGHV genes [45]. Another study also showed a high risk of skin cancer development in CLL patients with absent IGHV mutations, probably due to the more profound immunosuppression associated with the aggressive, IGHV-unmutated CLLs [46]. 

The Protection of Telomers 1 (POT1) gene encodes a protein responsible for telomere protection, and its germline variants are associated with an increased risk of melanoma and have also been associated with familial CLL [47,48]. The POT1 tumor predisposition syndrome is a rare autosomal dominant familial cancer predisposition syndrome that carries a high risk of multiple primary melanomas, angiosarcoma, gliomas, and CLL [49]. Furthermore, somatic mutations of the POT1 gene are associated with melanoma (4% of sporadic melanomas) and CLL (3–7% of the cases) [50]. 

V-Raf murine sarcoma viral oncogene homolog B1 (BRAF) mutations are key genetic alterations in melanoma. Approximately 50% of melanoma patients carry somatic mutations in this gene, leading to the constitutive activation of the MAPK (mitogen-activated protein kinase) pathway and accelerated cell proliferation, increased cell survival, and invasion [51]. BRAF mutations are detected in almost all cases of hairy cell leukemia, namely the BRAF V600E mutation [52]. In CLL, BRAF mutations are found with a low frequency (2.8% of cases) in a study conducted by Jebaraj et al. [53]. BRAF mutations were present in 2% of CLL cases in another series and involved other mutations than the V600E mutation [54]. Sellar et al. [55] observed a higher frequency of the BRAF V600E mutation in CLL patients with Richter transformation. 

##### Therapeutic Factors

The treatment of CLL might be another contributing factor, mainly due to its immunosuppressive effect. Several studies found higher rates of melanoma in patients who underwent chemotherapy [18,56]. Lam and colleagues [57] observed a higher frequency of melanoma development after the treatment of CLL/SLL with chemotherapeutic regimens containing fludarabine (hazard ratio 1.90; 95% CI, 1.08–3.37) compared to patients who did not undergo chemotherapy or who were treated only with oral therapeutic agents. Moreover, the most extensive non-registry-based international study showed that chemotherapy with fludarabine and cyclophosphamide with or without rituximab was associated with melanoma development in the univariable analysis. However, most patients who needed treatment for CLL had an unmutated IGHV status, which was already associated with the risk of melanoma [45]. Some of the studies mentioned above also investigated the influence of therapeutic factors on melanoma development in CLL/SLL survivors, a detailed summary of which is presented in Table 3. 

##### Surveillance Bias

Finally, surveillance bias could also contribute to the higher rate of melanoma diagnosis in CLL patients, as these patients undergo regular clinical examinations. The role of surveillance bias is supported by the more frequent occurrence of melanoma in the first few years (2–5 years) after CLL diagnoses [18,19,30,31,57]. 

#### 3.1.2. Impact on Survival

CLL/SLL patients have higher mortality compared to the general population, which is significantly increased by the occurrence of a second cancer, to which melanoma contributes. In the study by Royle et al. [27], melanoma increased the risk of death in CLL patients almost five-fold, and the authors reported a standardized mortality ratio (SMR) for melanoma of 4.79 (3.83–5.90). They suggested that melanoma might have a more aggressive behavior in the context of CLL. Similarly, Herr et al. [19] demonstrated an increased risk of death in patients with melanoma after CLL, regardless of the cause. Moreover, melanoma survival is negatively influenced by the presence of a history of CLL, with these patients having an odds of death 1.46 higher than patients without CLL [20]. A retrospective case-control study analyzed the impact of a personal history of CLL on melanoma mortality and recurrence, demonstrating significantly higher mortality due to melanoma (hazard ratio 2.46 (95% CI, 1.27–4.74)) and a significantly higher risk of melanoma recurrence (hazard ratio 3.44 (95% CI, 1.79–6.63)) [58]. A large population-based cohort study conducted by Brewer et al. [59] showed a worse prognosis for melanoma patients who also had CLL, with an SMR of 2.76 (95% CI, 2.22–3.44). The authors indicated a significantly worse melanoma-specific survival for any Breslow thickness equal to or above 1 mm. Therefore, CLL not only contributes to the risk of melanoma development but also exacerbates melanoma prognosis, both at least partly attributable to the altered immune function. 

#### 3.1.3. Therapeutic Challenges

The management of advanced-stage melanoma is a challenge, especially in the context of coexisting CLL, due to the scarcity of data regarding the effectiveness and safety of immunotherapy for advanced melanoma in patients with concomitant hematologic malignancies, as these patients were excluded from the clinical trials. Due to the underlying immune dysfunction, CLL patients are at a higher risk of immune-mediated diseases and might have a poorer tolerability of immunotherapy [58]. 

A retrospective case series of 15 patients with coexisting advanced melanoma and CLL did not show an altered response to immunotherapy (ipilimumab, pembrolizumab, and ipilimumab + nivolumab) due to the concomitant lymphoproliferative disease and effectiveness was not decreased in the presence of CLL. In the same series, severe adverse events (grade 3 and 4) occurred with ipilimumab in 50% of patients, while these are reported to occur in only 15–28% of patients with no associated neoplasia [60]. A larger study based on data from the Dutch Melanoma Treatment Registry analyzed 4638 advanced melanoma cases who underwent anti-programmed cell death-1 (anti-PD-1) monotherapy, ipilimumab-nivolumab therapy, or targeted therapy with BRAF/Mitogen-activated protein kinase (MEK) inhibitors, among which 46 patients from the anti-PD-1 therapy group, 11 patients from the ipilimumab-nivolumab group, and 43 patients from the BRAF/MEK inhibitor group had a concomitant hematologic malignancy. This study showed a significantly worse median progression-free survival in patients with associated hematologic neoplasia treated with anti-PD-1 therapy compared to patients without concomitant malignancies (2.8 months versus 9.9 months) and significantly shorter melanoma-specific survival (41.2 months versus 58.1 months). The results were similar for ipilimumab-nivolumab therapy, with a median progression-free survival of 2.3 months in patients with concomitant hematologic malignancy compared to 6.8 months for patients without associated cancer and a median melanoma-specific survival of 4.6 months versus 46 months. Outcomes for the patients treated with BRAF/MEK inhibitors were not significantly different between patients with a concomitant presence/absence of a hematologic malignancy. However, this study did not evaluate the efficacy of these therapies, specifically in patients with CLL/SLL, but in patients with various types of hematologic malignancies [61]. Still, data are by far insufficient, and until more extensive cohort studies or randomized controlled trials are available, no reliable conclusions can be formulated.

Considering the worse outcomes and shorter survival of patients with melanoma and CLL and the lack of data for the management of this particular subgroup of melanoma patients, prevention and early detection might be the best strategy to improve survival. William et al. [62] conducted a cohort study including 470 CLL patients with 22 newly developed melanomas, of which 15 cases were diagnosed due to active screening in a dermatology clinic and 2 in a lymphoma clinic. A total of 88.2% of the actively discovered lesions were not in an advanced stage at diagnosis, highlighting the potential role of active surveillance in these patients. Therefore, all patients with a diagnosis of CLL may benefit from active skin cancer screening to detect lesions at an early stage and improve prognosis. Moreover, all patients should receive sun protection education and be trained in skin self-examination. Primary health care providers should also be warned about the higher risk of melanoma in these patients to increase the index of suspicion and encourage referral to specialists in the early recognition of melanoma. Skin surveillance is essential not only because of the risk of melanoma but also because of the high risk of other types of skin cancer (squamous cell carcinoma, basal cell carcinoma, Merkel cell carcinoma, and Kaposi sarcoma) [21,36]. Although beyond the scope of this review, it must be highlighted that the association between CLL and melanoma is mutual, as melanoma patients also present a higher risk of CLL development compared to the general population [19].

### 3.2. Melanoma as Second Primary Cancer after Non-Hodgkin Lymphoma

Worldwide, there were 544,000 new cases of non-Hodgkin lymphoma (NHL) reported in 2020, according to GLOBOCAN [1]. The incidence rates of NHL have increased in the last decades and are estimated to continue to rise in the next two decades by 43% [63]. Although advances in treatment led to improved survival rates [64], survivors of NHL have an increased risk of developing second primary tumors at multiple sites, including other hematologic cancers and solid organ neoplasia [65]. Melanoma as a second primary cancer following NHL has repeatedly been reported in large population-based studies, as shown in Table 4.

Non-Hodgkin lymphoma comprises a large group of different subtypes, some of them with an indolent, others with an aggressive course. 

In the study of Chattopadhyay et al. [70], the risk of melanoma was increased after all frequent subtypes of NHL, including (DLBCL) diffuse large B-cell lymphoma (RR 1.58), (FL) follicular lymphoma (RR 2.28), (LPL) lymphoplasmacytic lymphoma (RR 2.04), (MCL) mantle cell lymphoma (RR 2.84), and (MZL) marginal zone lymphoma (RR 5.85), whereas among the rarer subtypes, melanoma risk was significantly increased only after (BL) Burkitt lymphoma (RR 4.77). Another study conducted by Morton et al. [28] demonstrated an elevated risk of melanoma after follicular lymphoma and CLL/SLL but not after diffuse large B-cell lymphoma. In the cohort of Giri et al. [75], 77 melanoma cases occurred in 1540 follicular lymphoma patients, the most common indolent NHL subtype, with an observed-to-expected ratio of 1.38 (95% CI, 1.09–1.73). Herr et al. [19] analyzed the association between melanoma and different types of lymphoid neoplasms. They found that among the common types of NHL, patients with DLBCL (SIR 1.22, 95% CI, 1.02–1.45) and FL (SIR 1.32, 95% CI, 1.09–1.58) had a statistically significant increased risk of melanoma. Patients with MZL also presented an elevated risk, albeit not statistically significant. 

Sex differences are discordant between the studies. In the study of Goggins et al. [22], the risk of melanoma after NHL was similar in men and women. In contrast, in the study of Hall et al. [72], the risk was significantly elevated only in women. Lam et al. [57] and Travis et al. [65] reported a higher risk among male patients.

Regarding the timeframe in which melanoma developed, the risk was highest in the early period after NHL diagnosis (first year—RR = 3.38) and then decreased slowly (1–5 years—RR = 2.25; 6–10 years—RR = 2.0; 11–20 years—RR = 1.96) but remained elevated, even slightly increased after 20 years (>20 years—RR = 2.05) in the study of Bermejo et al. [68]. Royle et al. [74] showed the maximum risk after 9 years from NHL diagnosis (SIR 5.88). Goggins W et al. [22] observed the most significant risk in the first 3 years, followed by a decrease in the risk and a new peak after 10 years, whereas Hall P et al. [72] found the highest risk 3 to 10 years after an NHL diagnosis. 

#### Factors Leading to Increased Melanoma Risk in NHL Survivors

Speculations about possible explanations for the association between melanoma and NHL include an altered immune response, shared genetic and environmental risk factors, treatment-related factors [76], and possibly increased surveillance.

The presence of a bidirectional association between the two malignancies, as shown in the literature [22,66], would point towards shared genetic and/or environmental risk factors. Sun exposure seems to have opposite roles in melanoma and NHL development.

A meta-analysis conducted by Kim and Kim [77] demonstrated a mild/moderate protective role of UV radiation for NHL for both personal and ambient exposure. Among the subtypes of NHL, sun exposure was a protective factor for all subtypes except T-cell lymphoma (personal exposure) and CLL/SLL (ambient exposure). Intense intermittent exposure had a higher protective role, whereas occupational exposure was only associated with a lower risk of DLBCL and FL. However, many of the included studies were retrospective, and considerable heterogeneity existed between them.

These data argue against UV radiation as a shared environmental risk factor for both malignancies. However, sun exposure is the strongest environmental risk factor for melanoma, and its oncogenic effects might be amplified in the context of disease-related and/or therapy-related immunosuppression.

Immunosuppression, especially therapy-related, might also be responsible [65]. The timeframe of melanoma development with a higher frequency of melanoma early after an NHL diagnosis suggests a possible implication of surveillance bias but also shared risk factors. However, the increase in risk, even after ten years, favors the involvement of treatment-related effects [22]. Lam et al. [57] explored risk factors for melanoma development following different types of NHL. The authors revealed no association between the risk of melanoma and the treatment for NHL other than CLL/SLL. However, the number of non-CLL/SLL NHL patients who developed melanoma was relatively small in the cohort.

A large population-based study assessed the risk of second primary neoplasia after autologous hematopoietic cell transplantations in 7765 patients, among which 3107 (40%) received this treatment for the indication of NHL. Among the 298 second primary tumors, melanoma was the most frequent solid neoplasia, with a significant risk in the first four years after intervention and in patients who received treatment for either NHL or multiple myeloma. The SIR for melanoma development after autologous hematopoietic cell transplantation for the indication of NHL was 2.66 (95% CI, 1.80–3.94). The authors also demonstrated a higher risk among older patients as well as in men and argued that the risk of melanoma in these patients might be attributed to the higher sun exposure accumulated throughout the years and the immune imbalance due to chemotherapy and transplantation [78]. In a large multi-institutional cohort study including 28,000 patients, melanoma occurred more frequently in patients who underwent allogenic hematopoietic cell transplantation, with an observed-to-expected ratio of 3.47 (95% CI, 2.06–5.49) but in this study, the proportion of patients who received the transplant for the indication of NHL was only a small proportion (4%) [79].

Case reports raised questions about a possible association between melanoma development or progression and treatment with rituximab, an anti-CD20 monoclonal antibody often used in the treatment of NHL [80,81,82]. However, in the context of NHL, which is already associated with melanoma risk, it is difficult to assess whether rituximab has played a role in melanoma development or progression. Cengiz et al. [83] conducted a dermoscopic one-year follow-up study in 16 rituximab-treated patients, among which 15 received this treatment for NHL and 1 for pemphigus vulgaris. The authors observed that rituximab therapy was associated with morphological changes in nevi (26% of nevi increased in size, and 17% developed atypical dots and clods), but none of the patients developed melanoma. A meta-analysis of randomized controlled trials explored the risk of second primary cancer in lymphoma patients treated with therapeutic regimens with rituximab (2312 patients) and without rituximab (2309 patients). The authors did not demonstrate an increased risk of second primary cancers associated with rituximab therapy [84].

### 3.3. Melanoma Following Hodgkin Lymphoma

Hodgkin lymphoma (HL) is a hematologic malignancy with a global age-standardized incidence rate of 0.98/100,000 persons [85] and two incidence peaks in early adulthood and adults older than 55 years of age [86]. Hodgkin’s disease presents a good prognosis, with an overall survival above 90% in the early disease and 75–90% in advanced stages [87]. Survivors of HL are at an elevated risk of second primary neoplasia, either other hematologic malignancies or solid tumors [88], which can impact prognosis. Although the risk of some second cancers decreased over time in younger patients (breast cancer and gastric cancer), primarily due to improved therapeutic regimens, the risk of most second cancers remained elevated [89]. 

Melanoma risk was found to be increased in several studies assessing the risk of second primary malignancies in HL survivors, as presented in Table 5.

Regarding the timeframe of melanoma occurrence after HL, in most studies, the risk was significant only in the first five years after HL treatment or was more important in this early period [19,90,97]. However, in the study of Royle et al. [74], the risk of melanoma after HL was highest more than nine years after HL diagnosis (SIR 18.9). The same study also showed that melanoma risk was most significant for patients who were younger at first diagnosis (younger than 40 years of age, SIR 19.7). Another study did not observe differences regarding the age of the patients (melanoma risk was similar for patients younger or older than 40 years of age) [98]. Sex differences were observed in the studies; Dores et al. [88] demonstrated a statistically significant elevated risk of melanoma among male patients (O/E ratio 2.2 in men versus 1.1 in women). In contrast, the risk was elevated only in female patients in the study by Abrahao et al. [98].

Melanoma following HL might be associated with the treatment applied for the first neoplasm. Although melanoma is not strongly associated with radiotherapy, in the study conducted by Abrahamsen et al. [90], among the eight observed cases of melanoma, seven developed in patients who received radiotherapy alone and one in a patient who followed combined treatment with radiotherapy and chemotherapy. In contrast, none developed in patients who were treated with chemotherapy only. Additionally, six of the melanomas appeared within the irradiation field. In a cohort study conducted by Swerdlow et al. [99], the risk of melanoma development following HL was only statistically significant in the group of patients who underwent combined treatment with chemotherapy and radiotherapy (SIR 2.7, 95% CI 1.1–5.5, *p* < 0.5). In contrast, patients who followed chemotherapy alone had no significant risk of melanoma (SIR 0.5, 95% CI 0.01–2.8). 

Although there is not enough data to suggest that radiotherapy increases melanoma risk in HL survivors, it is worth discussing that modern radiotherapy techniques could reduce this risk. The introduction of newer approaches, such as involved-site and involved-node radiation therapy, which aim to reduce the volume of irradiated tissue, can significantly reduce the long-term toxicity of this treatment and, thus, the second cancer risk. The second cancer risk reduction with these techniques used in the treatment of HL has been demonstrated for other cancers, such as breast, lung, and thyroid [100]. A recent randomized multicenter phase III study has shown that in early unfavorable Hodgkin lymphoma, involved-site radiotherapy as consolidation therapy following combined chemotherapy was not significantly different in progression-free survival compared to the traditional involved-field radiation therapy. Moreover, the study showed that with involved-site radiation therapy, the acute toxicity could be reduced compared to involved-field radiation, which can also be extrapolated into reduced long-term toxicity, including reduced second cancer risk [101]. Further advances in radiotherapy include image-guided radiation therapy (IGRT) techniques, using computed tomography, positron emission tomography, magnetic resonance imaging, or echography, which aim to improve the visualization of the target tissue and precise irradiation of the tumor despite the physiological changes in tissue volume and location [102]. A recent case report showed the benefits of magnetic resonance imaging-guided respiratory-gated intensity-modulated radiotherapy in reducing the irradiation to the tumor-surrounding healthy tissue in the case of a large paravertebral rhabdomyosarcoma metastasis. This technique offered a better display of the anatomy of the surrounding soft tissues and led to a better optimization of the dose to organs at risk [103]. Although there are no data investigating the reduction in melanoma risk with different radiotherapy techniques, we could assume that these newer approaches could also reduce melanoma second cancer risk.

It is also possible that the intensive chemotherapy often used for the treatment of HL could contribute to melanoma development due to immune perturbations [19]. An interesting finding of the study conducted by Abrahao et al. [98] was that among HL survivors, the risk of melanoma development was only elevated for patients without an HIV infection, whereas HIV-infected patients had no significant risk. 

It has been observed that the risk of melanoma is higher in the initial years after being diagnosed with HL. This suggests that the possibility of surveillance bias cannot be ruled out. In a Swedish study conducted by Don and Hemminki [71], the risk of melanoma was highest in the first year after HL diagnosis (SIR 6.65), followed by the 1–9 year interval (SIR 2.29). After nine years, there was no significant risk increase, which further supports the theory of surveillance bias.

Melanoma increases the risk of death in HL patients from any cause (hazard ratio 2.46, 95% CI, 1.45–4.16) [19]. The increased awareness of melanoma risk in these patients could lead to an earlier diagnosis and reduced mortality. 

### 3.4. Melanoma Following Primary Cutaneous Lymphoma

Primary cutaneous lymphomas are the second most frequent extra-nodal non-Hodgkin lymphomas and comprise a heterogeneous group of lymphoproliferative malignancies that primarily affect the skin without involvement of extracutaneous sites in the initial stages [104]. Most primary cutaneous lymphomas are of T-cell origin (65%), while others originate from B-lymphocytes (25%) or natural killer cells (NK cells) [105]. 

Mycosis fungoides (MF) is the most common type of cutaneous T-cell lymphoma, with an increasing incidence of almost 3-fold compared to the 1980s. In general, MF presents a good prognosis, depending on the stage (5-year overall survival of 91–97% for limited involvement, 81–85% for generalized disease, 44% for tumoral stage, 20–30% for lymph node involvement, and only 38,5% for transformed MF) [106]. 

A large population-based study performed using the SEER-18 data has shown an increased risk of second primary cancers in patients with MF (6742 patients, 511 s cancers, SIR 10.15, 95% CI, 9.29–11.07), including non-Hodgkin and Hodgkin lymphoma, melanoma, pulmonary, breast, prostate, colon, and renal cancer. In this study, the risk of melanoma after one year from the diagnosis of MF was significantly elevated (SIR 9.0, 95% CI, 5.50–13.90) [107]. Another population-based cohort study (SEER-9) also demonstrated a significantly increased risk of melanoma after MF (SIR 2.60, 95% CI, 1.25–4.79) [108]. A single-center study including 672 patients with MF demonstrated an increased second cancer risk, although, for melanoma, the risk was not statistically significant [109]. In another study, based on SEER-13 data, the risk of melanoma following MF was increased but without statistical significance [110]. A Danish population-based cohort study found only one case of subsequent melanoma among 386 MF patients in a mean follow-up period of 7.6 years [111]. A study in Israel analyzed the risk of melanoma development in patients with MF (n = 982) and compared it with the melanoma risk of both psoriasis patients (n = 3164) and the general population. The authors demonstrated a significantly high risk of melanoma in MF patients (SIR = 17.5, 95% CI 11.0–23.9 *p* < 0.0001) as compared to the matched general population, while the SIR was not elevated for patients with psoriasis (SIR 2.2, 95% CI 0.6–3.8, *p* = 0.0148). Moreover, the authors evaluated phototherapy as a risk factor for melanoma development in MF and psoriasis patients and found no significant associations [112]. 

Licata et al. [113] investigated the risk of second skin cancer after total skin electron beam radiation for MF. They found not only a higher risk of non-melanoma skin cancer but also a higher risk of melanoma, with 5% of patients developing melanoma in a median time of 35 months after this therapy. The authors also evaluated the effects of other treatments, mechlorethamine or PUVA (oral psoralen plus ultraviolet A), but failed to demonstrate a significant association with the risk of melanoma. However, the number of patients included in this study was relatively low. 

A longitudinal study followed 197 MF patients for second malignancy and evaluated the effects of different treatment approaches. Of 151 patients treated with radiotherapy (including 104 patients with total skin electron beam irradiation), 8 patients developed a subsequent melanoma in contrast to only 1 patient among those who did not receive radiation treatment (46 patients) [114]. 

Lindahl et al. [115] evaluated the hypothesis that topical nitrogen mustard might increase the risk of second cancers in MF patients. They showed no increased risk of melanoma and other cancers in 110 patients treated with topical nitrogen mustard compared to 193 patients who did not receive this treatment. 

Other than treatment-related effects might also play a role in melanoma risk in MF patients, as melanoma has also been reported to occur in MF patients not receiving potentially carcinogenic therapies. In a retrospective study that included 285 MF cases, four patients developed melanoma after the diagnosis of MF, among which only two had followed treatments other than topical corticosteroids [116]. In another study, melanoma developed in two MF patients who did not undergo specific skin-directed therapies that could be related to an increase in melanoma risk [117]. 

The hypothesis of immunosuppression may partly explain the higher risk of melanoma in MF patients, especially in advanced-stage disease. However, the early-stage disease is also characterized by local immune dysfunction. Martinez-Escala et al. [118] reported four young patients who developed multiple melanocytic nevi predominantly within the MF lesions. The authors argued that the altered cellular immune response in MF and the skin-directed therapy-related immune dysfunction might explain this. The role of UV was also discussed. Although the patients received whole skin phototherapy, they developed the melanocytic nevi only within the MF lesions, further underlining the role of local immunosuppression. 

The hypothesis of shared genetic risk could be supported by some mutual genetic abnormalities, such as abnormalities in the p15 and p16 genes, which have been reported to be associated with a third of familial melanoma cases. P15 and P16 gene abnormalities were detected in 57% and 45% of MF or Sezary syndrome patients [119]. 

Regarding environmental risk factors, UV exposure is a well-established risk factor for melanoma, but its role in MF is still debatable. Gniadecki et al. [120] analyzed the frequency of UV signature mutations in the lesional skin of MF patients. They found a very low frequency of these mutations in MF (<5%), arguing against the possible role of UV exposure in MF development. 

Just as importantly, melanoma might be diagnosed more frequently among MF patients compared to the general population because they undergo more frequent and detailed skin examinations. Thus, surveillance bias cannot be ruled out. 

### 3.5. Melanoma Following Multiple Myeloma

Multiple myeloma (MM) is a plasma cell neoplasm with an increasing incidence in developed countries, albeit with improved survival in the last decades [121]. Due to the immunosuppression or treatment-related effects, multiple myeloma patients might be predisposed to develop second primary cancers, especially secondary hematologic neoplasms, but also solid tumors, although with a much lower incidence [122]. Data regarding the risk of second primary melanoma following MM are heterogeneous, with most studies showing no increased risk of melanoma in these patients. In a retrospective cohort study including 205 MM patients and 193 controls, the risk of melanoma was not statistically significant (SIR 1.69, 95% CI, 0.83–3.11, *p* = 0.109). However, the number of patients included in the study was relatively small [123]. A large population-based study using the SEER data also failed to demonstrate a significantly higher-than-expected risk of melanoma among MM patients compared to the general population [124]. Another large cohort study based on the data from the Cancer Registry of Norway included 9574 MM patients and 37,810 matched controls and showed a non-increased risk of melanoma following MM (hazard ratio 0.93, 95% CI, 0.56–1.53) [125]. In contrast, a large SEER-based study showed a higher risk of melanoma in MM patients than expected in the general population (SIR 1.36, 95% CI 1.07–1.74) [126]. Mahindra et al. [127] evaluated the risk of second primary cancers in 4161 MM patients who underwent autologous transplants. The authors demonstrated an increased risk of melanoma with a statistically significant observed-to-expected ratio of 3.58 (99% CI, 1.82–6.29), similar to other transplant recipients. However, melanoma in this context may be due to transplant-related effects rather than the presence of MM.

### 3.6. Melanoma Following Philadelphia Chromosome-Negative Myeloproliferative Disorders

Philadelphia chromosome-negative myeloproliferative disorders are clonal hematologic neoplasms, which include polycythemia vera (PV), essential thrombocythemia (ET), and idiopathic myelofibrosis (IMF). PV and ET are characterized by a long evolution with a survival of often more than 15 years. However, IMF presents a worse prognosis, with a median survival of five years [128]. Patients with Philadelphia chromosome-negative myeloproliferative neoplasms have been repeatedly reported to be at risk of developing second primary neoplasms, both hematologic and solid cancers [129,130]. A summary of the literature regarding the risk of melanoma as a second primary cancer following Philadelphia chromosome-negative myeloproliferative disorders is presented in Table 6.

### 3.7. Melanoma Following Hematopoietic Stem Cell Transplantation

Melanoma occurring after a hematopoietic stem cell transplant (HSCT) is highly relevant for this literature review, as HSCT is an essential component of the complex management of patients with hematologic malignancies. There is a growing body of evidence that indicates a high risk of melanoma development after different types of HSCTs for various hematologic malignancies. 

Curtis RE et al. [135] investigated the risk of new solid tumors after allogenic and syngeneic transplants in a large cohort of 12,229 patients, among which most patients have undergone this treatment for the indication of leukemia, and most of them have received it from an HLA-identical family member. The authors observed a significantly increased risk of melanoma development (observed-to-expected ratio 5.0 (95% CI, 2.5–8.9, *p* < 0.05)), among other cancers. In a single-center cohort in Finland, which included 1179 patients who received an allogeneic stem cell transplant, melanoma was the most frequent second cancer, with an SIR of 8.9 [136]. In another large European cohort study, 4065 patients developed a second cancer from the 220,617 transplant patients, where melanoma was among the five most frequently occurring second cancers (343 patients) [137]. A Danish population-based study showed an increased risk of melanoma after an allogenic HSCT (hazard ratio 5.5 (95% CI, 1.7–17.7, *p* = 0.04)), while the risk for patients receiving an autologous HSCT was not elevated. Moreover, the authors also demonstrated that allogenic HSCT recipients had a three-fold increased risk of melanoma compared to renal transplant patients, as opposed to the risk of squamous cell carcinoma, which is significantly higher in renal transplant recipients [138]. Another study, which investigated melanoma risk among patients with immunosuppression of various causes, also found an elevated risk of melanoma in bone marrow transplanted patients, both autologous and allogenic, with an SIR of 2.90 (95% CI, 1.33–5.51) and SIR of 3.18 (95% CI, 1.128–6.56), respectively. In this cohort, the SIR was also higher in bone marrow transplant patients than in solid-organ transplant patients (SIR 1.21; 95% CI, 0.82–1.73). However, the authors concluded that the difference was due to the higher mortality of bone marrow transplant patients in the post-transplant period and to the smaller number of patients [139]. In the study of Arora M et al. [140], including 630 multiple myeloma patients who had undergone autologous blood or bone marrow transplantation, 42 patients developed a subsequent tumor, among which melanoma (11 cases) along with cutaneous squamous cell carcinoma (11 cases) were the most common second malignancies.

Few studies investigated the risk factors for melanoma development after an HSCT. In their cohort of allogenic HSCT patients, Curtis RE et al. [135] found a higher risk of melanoma in patients who received total-body irradiation (TBI) in high doses and in patients who received T-cell-depleted donor grafts. Herr MM et al. [141] evaluated risk factors for melanoma in allogenic HCST patients in a case-control study, which included 140 melanoma patients and 557 matched controls. Risk factors for melanoma in these patients were living in a geographic location with higher ambient ultraviolet radiation at the time of intervention, the receipt of myeloablative conditioning with total body irradiation or reduced intensity conditioning with melphalan or fludarabine (versus busulfan-based conditioning), the development of acute graft-versus-host disease with stage 2+ cutaneous implication or chronic graft-versus-host disease without cutaneous involvement, and having developed a keratinocyte carcinoma. In contrast to the study of Curtis RE et al. [135], the depletion of T-cells from the graft was not associated with higher melanoma risk. The association of melanoma with total body irradiation is unexpected, as melanoma risk is not generally associated with exposure to ionizing radiation. A detailed analysis of radiation exposure and skin cancer risk highlighted that although low-to-moderate radiation doses are not associated with increased melanoma risk, radiotherapy for malignant diseases or in the context of bone marrow transplant possibly has a different relationship with melanoma. It is still not known whether melanoma is directly associated with radiotherapy in this context or whether it is related to other factors, such as increased surveillance, the combined effects of radiotherapy with chemotherapy, immunosuppression caused by the treatment, or the underlying hematologic malignancy itself. In any case, insufficient data are available to support the idea that radiation increases melanoma risk [142].

## 4. Implications for Melanoma Screening Recommendations

There is still controversy about melanoma screening recommendations, but most organizations recommend melanoma screening for high-risk individuals who have risk factors for melanoma [143]. Hepner et al. [144] proposed that survivors of CLL and lymphoma should be included in the high-risk category, for whom melanoma screening is recommended. A German questionnaire-based observational study evaluated the frequency of cancer screening among adult hematologic cancer survivors. In this study, 70.1% of patients who responded to the questionnaire underwent skin cancer screening provided by an oncologist or a primary care physician, with a higher percentage among patients who received allogeneic transplantation [145]. However, there are no available data regarding the frequency of skin cancer screening in these patients from other countries.

Patients with hematologic cancers are at a higher risk of developing melanoma, which makes regular skin examinations necessary. Unlike screening procedures for other types of cancer, skin cancer screening is non-invasive and less expensive. Moreover, patients should be encouraged to perform regular skin self-examinations and seek specialist help if they notice a new or changing skin lesion.

## 5. Conclusions

Survival rates for hematologic cancer are improving. However, survivors face an increased risk of second cancers, including melanoma. Data showed that the highest risk concerns survivors of chronic lymphocytic leukemia and non-Hodgkin lymphoma, but it may also be significant for Hodgkin lymphoma, primary cutaneous T-cell lymphoma, and Philadelphia-chromosome negative myeloproliferative neoplasms.

The underlying mechanisms of these associations still need to be explained. However, the secondary immune deficiency associated with these neoplasms, treatment toxicity, a potential shared genetic predisposition, and environmental factors might all contribute to this risk. Further research into the genetic basis of these associations and the risk factors for melanoma in these patients could provide opportunities for the early identification of patients at risk.

As melanoma increases the risk of death in survivors of several types of hematologic neoplasia, it is crucial to take proactive measures to reduce this risk. Improved skin surveillance can lead to earlier melanoma detection and better survival outcomes. Primary care physicians should know this risk and encourage patients to use sun protection and skin self-examination. Developing screening programs and guidelines for dermatologic monitoring could enhance health outcomes for these patients.

## Figures and Tables

**Table 1:** A summary of the relevant population-based studies regarding the risk of melanoma as a second primary cancer after chronic lymphocytic leukemia/small cell lymphoma.

Authors	Year	Country/Registry	First Neoplasm	No Patients Followed	O	SIR (95% CI)	Follow-Up
Brewer et al. [21]	2015	USA/SEER 13	CLL	28,964	268	2.0 (1.8–2.2)	PYAR, mean ± SD 4.7 ± 3.8
Herr et al. [19]	2018	USA/SEER 17	CLL/SLL	36,784	287	1.96 (1.74–2.21)	Mean FU 3.3–6 yrs
Hisada et al. [26]	2001	USA/SEER	CLL	16,367	90	3.18 (NR)	84,667 PYFU
McKenna et al. [30]	2003	Scotland/ SCR	CLL	4016	6	2.3 (0.0–2.4) n.s	14,450 PYFU
Morton et al. [28]	2010	USA/SEER 11	CLL/SLL	15,915	85	1.92 (1.53–2.37)	4.3 Mean PYFU
Royle et al. [27]	2011	Australia/NCSCH	CLL	13,580	272	7.74 (6.85–8.72)	75,878 PYFU
Schöllkopf et al. [29]	2007	Denmark/DCR	CLL	12,373	27	2.42 (1.66–3.53)	47,636 PYFU
van der Straten et al. [31]	2023	Netherlands/NCR	CLL	24,815	278	2.74 (2.43–3.08)	162,698 PYFU
Turk et al. [18]	2019	USA/SEER 18	CLL	48,876	474	2.07 (1.89–2.27)	ns

USA—United States of America; SEER—Surveillance, Epidemiology, and End Results; NCSCH—National Cancer Statistics Clearing House; CLL—Chronic lymphocytic leukemia; SLL—small cell lymphoma; O—number of observed cases; SIR—Standardized incidence ratio; CI—Confidence interval; PYFU—person-years of follow-up; FU—follow-up; yrs—years; DCR—Danish Cancer Registry; SCR—Scottish Cancer Registry, n.s—not statistically significant, NCR—Netherlands Cancer Registry; PYAR—person-years at risk; SD—standard deviation, ns—not specified.

**Table 2:** Sex differences in melanoma risk after chronic lymphocytic leukemia/small cell lymphoma.

Authors	Year of Publication	SIR in Men	SIR in Women
Brewer et al. [21]	2015	2.0	1.9
Herr et al. [19]	2018	2.01	1.81
Hisada et al. [26]	2001	3.14	3.28
McKenna et al. [30]	2003	2.9	1.6
Royle et al. [27]	2011	7.40	6.40
Van der Straten et al. [31]	2023	2.80	2.63

SIR—standardized incidence ratio.

**Table 3:** Therapeutic influences on melanoma development following CLL/SLL.

Authors	N	No Receiving Ch/R	No of MSC	No (%) of MSC with/without Ch/R			Melanoma Risk
				Ch	R ± Ch	No Tx	
Herr et al. [19]	36.784	7798/478	287	53 (18.4)	<5	234 (81.5)	ns
Morton et al. [28]	15.915	4.680/490	85	26 (30.5)	<3	59 (69.5)	ns
Turk et al. [18]	48.876	7827/0	474	70 (14.8%)	0	404 (85.2)	O/E 2.28 (95% CI, 1.77–2.88)
Lam et al. [57]	13.950	5.051/1.025	91	47 § (51.7)	<10	52 (57.1)	# HR 1.90, 95 CI 1.08–3.37)
Chatzikonstantinou et al. [45]	φ 19.705	7.128 */9	130	nos	nos	nos	τ OR 2.08 (95% CI, 1.51–2.87)

N—total number of patients included in the study; Ch—chemotherapy; R—radiotherapy; MSC—melanoma second cancer; Tx—treatment; ns—not significant difference; nos—not specified; §—includes the number of melanoma cases who received any fludarabine or cyclophosphamide; #—significant in patients who received chemotherapy with regimens containing fludarabine; φ—also includes MBL (high-count CLL-like monoclonal B-cell lymphocytosis); *—includes chemotherapy and chemoimmunotherapy; τ—significant for fludarabine + cyclophosphamide ± rituximab.

**Table 4:** A summary of the relevant population-based studies regarding the risk of melanoma as a second primary cancer after non-Hodgkin lymphoma.

Authors	Year	Country/Registry	No Patients	O	M Risk (95% CI)	FU Time
Adami et al. [66]	1995	Denmark + Sweden/Danish Cancer Registry	34,641	52	RR: 2.4 (1.8–3.2)	114,423 PY
Baras et al. [67]	2017	Germany/ZfKD	110,164	328	SIR: 1.92 (1.71–2.14)	462,890 PY
Bermejo et al. [68]	2014	Finland, Norway, Sweden	60,901	217	RR: 2.27 (1.98–2.59)	324,798 PY
Brennan et al. [69]	2020	Australia/NSW Central Cancer Registry	12,452	93	SIR: 2.38 (1.92–2.91)	54,308 PY
Chattopadhyay et al. [70]	2018	Sweden/Swedish FCD	19,833	83	RR: 1.98 (1.60–2.44)	4 y median FU
Dong and Hemminki [71]	2001	Swedish Family Cancer Database	18,960	33	1.14 (0.78–1.60) n.s	94,088 PYAR
Goggins et al. [22]	2021	USA/SEER	62,597	139	SIR: 1.75 (1.48–2.07)	m: 123,288 f: 121,288 PY
Hall et al. [72]	1995	Sweden/SCR	6176	10	SIR: 1.7 (0.8–3.1)	ns
Parsons et al. [73]	2023	USA/SEER	141,451	715	SIR: 1.29 (1.20–1.39)	923,475 PY
Royle et al. [74]	2011	NCSCH	40.529	313	SIR: 5.24 (4.67–5.85)	198,717 PYAR
Travis et al. [65]	1993	ICR + OCR + SCR + affiliated tumor registry of the NCI	6171	20	O/E: 2.38 (1.45–3.67)	7.4 y mean FU

M—melanoma; O—number of observed cases; SIR—standardized incidence ratio; CI—confidence interval; RR—relative risk; NHL—Non-Hodgkin lymphoma; ZfKD—German Center for Cancer Registry Data; PY—person-years of observation; USA—United States of America; SEER—Surveillance, Epidemiology, and End Results; NSW—New South Wales; Swedish FCD—Swedish Family-Cancer Database; y—years; FU—follow-up; m—male, f: female; SCR—Stockholm-Gotland Cancer Register; ns—not specified; ICR—State Health Registry of Iowa; OCR—Ontario Cancer Registry; NKI—Netherlands Cancer Institute; O/E: observed-to-expected ratio; NCSCH—National Cancer Statistics Clearing House, PYAT—person-years at risk; n.s—not statistically significant.

**Table 5:** A summary of the relevant studies regarding the risk of melanoma as a second primary cancer following Hodgkin lymphoma.

Authors	Year	Registry/Hospital	No Patients	M Risk (95% CI)	FU Time
Abrahamsen et al. [90]	2002	Norwegian Cancer Registry	1.024	SIR: 2.8 (1.2–5.5) ^#^	14 y median FU
Andrea et al. [91]	2002	Four Harvard affiliated hospitals ^+^	1319	RR 3.3 (1.3–6.7)	15.910 PYFU
Daniëls et al. [92]	2013	LUMC cancer registry system + PALGA + NCR	889	SIR: 2.3 (0.9–5.6)	ns
Dietrich et al. [93]	1994	Institut Gustave Roussy	892	O/E: 11.76 (1.42–43)	5.263 PYAR
Dong and Hemminki [71]	2001	Swedish Family Cancer Database	5353	1.83 (1.04–2.98)	46.206 PYAR
Henry-Amar et al. [94]	1992	IDHD	12.411	O/E m: 1.9 f: 1.2 n.s.	82.850 PYFU
Herr et al. [19]	2018	17 SEER Program registries	17.556	SIR: 1.75 (1.33–2.26)	3.3–6 y mean FU
Munker et al. [95]	1999	Munich tumor registry	1120	SIR: 2.5 (0.4–8.2)	9.1 y mean FU
Royle et al. [74]	2011	NCSCH	8.396	SIR: 8.00 (5.92–10.6)	68.369 PYAR
Sud et al. [96]	2017	Swedish Family-Cancer Project Database	9.522	SIR: 2.08 (1.54–2.82)	12.6 y median FU
Swerdlow et al. [97]	2000	BNLI database + Royal Marsden Hospital + St Bartholomew’s Hospital	5.519	SIR: 2.3 (0.9–4.6) n.s. SIR: 4.2 (1.3–9.9) ^#^	46.990 PYFU

M—melanoma; SIR—standardized incidence ratio; CI—confidence interval; RR—relative risk; HL—Hodgkin lymphoma; FU—follow-up; y—years; LUMC—Leiden University Medical Center; PALGA—Pathologisch-Anatomisch Landelijk Geautomatiseerd Archief; NCR—Netherlands Cancer Registry, ns—not specified; SEER—Surveillance, Epidemiology, and End Results; BNLI—British National Lymphoma Investigation; n.s.—not statistically significant; PYFU—person-years of follow-up; IDHD—International Database on Hodgkin’s Disease, m—male, f—female, O/E—observed/expected ratio; RR—relative risk; PYAR—person-years at risk; NCSCH—National Cancer Statistics Clearing House; ^#^ risk was statistically significantly elevated only in the period of the first five years after the first treatment/first five years of follow-up. ^+^ Brigham and Women’s Hospital, Dana-Farber Cancer Institute, Children’s Hospital, or Beth Israel Deaconess Medical Center.

**Table 6:** The risk of melanoma as a second primary cancer following Philadelphia chromosome-negative myeloproliferative disorders.

Authors	Year	Country/Registry	First Neoplasia	No Patients Followed	M Risk (95% CI)	Follow-Up
Chattopadhyay et al. [131]	2018	Sweden/ SFCD	PV ET MF MPN-nos	6.636 4081 1454 1634	RR 2.27 (1.65–3.14) RR 2.26 (1.50–3.40) RR 1.66 (0.62–4.42) n.s. RR 1.60 (0.72–3.56) n.s.	6 y median FU 4 y median FU 2 y median FU 3 y median FU
Fallah et al. [132]	2011	Sweden/ SCR	PV	3530	SIR 1.88 (1.13–2.93)	Not specified
Federiksen et al. [129]	2011	Denmark/DNRP	PV ET	4.625 1578	1.7 (1.0–2.7) NR	5 y median FU 4 y median FU
Joshi et al. [133]	2022	USA/ SEER 18	PMF	5.273	SIR 1.76 (1.01–2.86)	10 y
Landtblom et al. [130]	2018	Sweden/ SCR	PV, ET, PMF, MPN-U	c: 9.379 mc: 35.682	HR 1.9 (1.4–2.7)	7.7 y median FU
Susini et al. [134]	2012	Italy/UNIFI and RTT	PV, ET, MF	733	SIR 3.69 (1.39–9.64)	6.45 y mean FU

SCR—Swedish Cancer Register; PV—polycythemia vera; ET—essential thrombocythemia; (P)MF—(primary) myelofibrosis; MPN-U—unclassifiable myeloproliferative neoplasm; c—cases; mc: matched controls; M—melanoma; HR—hazard ratio; y—years; FU—follow-up; USA—United States of America; SEER—Surveillance, Epidemiology, and End Results; SFCD—Swedish Family-Cancer Database; MPN-nos—myeloproliferative neoplasm not otherwise specified; n.s.—not statistically significant; DNRP—Danish National Registry of Patients; NR—not reported; UNIFI—University of Florence; RTT—Tuscany Cancer Registry.
